# KDM5A silencing transcriptionally suppresses the FXYD3‐PI3K/AKT axis to inhibit angiogenesis in hepatocellular cancer via miR‐433 up‐regulation

**DOI:** 10.1111/jcmm.16371

**Published:** 2021-02-23

**Authors:** Yu‐Shui Ma, Ting‐Miao Wu, Bin Qian, Yu‐Shan Liu, Hua Ding, Ming‐Ming Fan, Ji‐Bin Liu, Fei Yu, Hui‐Min Wang, Yi Shi, Li‐Peng Gu, Liu Li, Lin‐Lin Tian, Pei‐Yao Wang, Gao‐Ren Wang, Zhi‐Jun Wu, Qi‐Fei Zou, Chang‐Chun Ling, Da Fu

**Affiliations:** ^1^ Central Laboratory for Medical Research Shanghai Tenth People's Hospital Tongji University School of Medicine Shanghai China; ^2^ Department of Radiology The Forth Affiliated Hospital of Anhui Medical University Hefei China; ^3^ Cancer Institute Nantong Tumor Hospital Nantong China; ^4^ Department of General Surgery Shanghai Eighth People's Hospital Shanghai China; ^5^ Department of Pathology Nantong Tumor Hospital Nantong China; ^6^ Department of Radiotherapy Nantong Tumor Hospital Nantong China; ^7^ Department of Biliary Surgery IV Eastern Hepatobiliary Surgery Hospital Shanghai China; ^8^ Department of Nuclear Medicine Shanghai Tenth People’s Hospital Tongji University School of Medicine Shanghai China; ^9^ Department of Oncology Nantong Second People's Hospital Nantong China; ^10^ Department of General Surgery The Affiliated Hospital of Nantong University Nantong China

**Keywords:** angiogenesis, FXYD domain‐containing ion transport regulator 3, FXYD3, invasion, microRNA‐433, migration, miR‐433, PI3K/AKT, proliferation

## Abstract

Hepatocellular cancer (HCC) has been reported to belong to one of the highly vascularized solid tumours accompanied with angiogenesis of human umbilical vein endothelial cells (HUVECs). KDM5A, an attractive drug target, plays a critical role in diverse physiological processes. Thus, this study aims to investigate its role in angiogenesis and underlying mechanisms in HCC. ChIP‐qPCR was utilized to validate enrichment of H3K4me3 and KDM5A on the promotor region of miR‐433, while dual luciferase assay was carried out to confirm the targeting relationship between miR‐433 and FXYD3. Scratch assay, transwell assay, Edu assay, pseudo‐tube formation assay and mice with xenografted tumours were conducted to investigate the physiological function of KDM5A‐miR‐433‐FXYD3‐PI3K‐AKT axis in the progression of HCC after loss‐ and gain‐function assays. KDM5A p‐p85 and p‐AKT were highly expressed but miR‐433 was down‐regulated in HCC tissues and cell lines. Depletion of KDM5A led to reduced migrative, invasive and proliferative capacities in HCC cells, including growth and a lowered HUVEC angiogenic capacity in vitro. Furthermore, KDM5A suppressed the expression of miR‐433 by demethylating H3K4me3 on its promoterregion. miR‐433 negatively targeted FXYD3. Depleting miR‐433 or re‐expressing FXYD3 restores the reduced migrative, invasive and proliferative capacities, and lowers the HUVEC angiogenic capacity caused by silencing KDM5A. Therefore, KDM5A silencing significantly suppresses HCC tumorigenesis in vivo, accompanied with down‐regulated miR‐433 and up‐regulated FXYD3‐PI3K‐AKT axis in tumour tissues. Lastly, KDM5A activates the FXYD3‐PI3K‐AKT axis to enhance angiogenesis in HCC by suppressing miR‐433.

## 1 INTRODUCTION

Hepatocellular cancer (HCC) is one of the most prevalent types of cancers with a high mortality rate worldwide.^1^ Hepatocellular cancer is likely to arise with longstanding chronic liver disease, such as chronic viral hepatitis types B and C, alcohol abuse, aflatoxin exposure and non‐alcoholic fatty liver disease^2^; viral hepatitis B and C still remain the main causative agents to the development of HCC.^3^ More importantly, HCC has been reported to belong to one of the highly vascularized solid tumours accompanied with hypervascularity and vascular abnormalities.^4^ It was also reported that transarterial chemoembolization (TACE) is one of the therapeutic approaches that improves the survival rates of patients with HCC; however, only a limited number of randomized controlled trials have been performed on assessing the survival benefit of patients undergoing TACE.^5^ Chemoresistance is still an ongoing issue that could induce the recurrence of disease.^6^ Anti‐angiogenic therapies have been utilized for HCC therapy, though the curative effect is very limited.^7^ Therefore, the knowledge of molecular mechanisms in angiogenesis is required for HCC therapy.

A majority of the human genome cannot encode proteins, which usually turn to be non‐coding RNAs such as microRNA, long non‐coding RNA or circular RNA.^8^ Increasing evidence demonstrates that miRNAs are implicated in the progression of HCC. For instance, miR‐338‐5p exerts inhibitory effects on the growth of HCC cells.^9^ Although the role of miR‐433 in HCC still remains exclusive, the physiological functions of miR‐433 in cancer progression have already been explored^10^; for instance, miR‐433 represses non‐small cell lung cancer progression by directly targeting smad2.^11^ In gastric cancer tissues, miR‐433 was found to be down‐regulated which corresponds to a poor outcome and a lower overall survival rate.^12^ To determine the upstream regulatory gene, ChIPBase database was used to predict the binding site between KDM5A and miR‐134. Epigenetic regulation is essential for the dynamic regulation of gene expression.^13^ The aberrant expressions of epigenetic modifiers are tightly associated with human diseases.^14^ H3K4me3, a marker of active gene expression, is the substrate of lysine demethylase 5A (KDM5A).^15^, ^16^ KDM5A, an oncogene and a promising drug target, has been reported to contribute to tumorigenesis, metastasis and drug resistance by repressing gene expression through demethylating H3K4me3.^17^, ^18^, ^19^ Moreover, a previous study has implied KDM5A being significantly up‐regulated in HCC.^20^ However, its role in HCC has not been well understood. A previous report demonstrated that FXYD3 was highly expressed in HCC tissues and was negatively correlated with overall survival rates.^12^ The potential binding sites between miR‐433 and FXYD domain‐containing ion transport regulator 3 (FXYD3) were predicted by Starbase. FXYD3 belongs to a family of Na, K‐ATPase regulators, which have a domain of FXYD.^21^ The family includes seven members, and different members are expressed in certain tissues where the seven members exert diverse functions.^22^ FXYD3 has been reported to be highly expressed in diverse cancer tissues and its aberrant up‐regulation contributes to the development of breast cancer by activating PI3K‐AKT signalling.^23^, ^24^ Aberrantly activated PI3K‐AKT is frequently found in diverse cancers and is correlated with a worse prognosis and outcome.^25^, ^26^ Constituted activation of PI3K‐AKT signalling may be attributed to the mutation occurring in PI3K or AKT, mutation or amplification of RTKs, or overexpression of other upstream genes.^23^, ^27^, ^28^ PI3K/Akt pathway could further induce multidrug resistance in HCC.^29^ Therefore, we hypothesize that KDM5A might be involved in HCC development by regulating the FXYD3‐PI3K‐AKT axis in an epigenetic manner through miR‐433.

## 2 MATERIALS AND METHODS

### 2.1 Ethical approval

The use of all specimens was agreed and approved by the Ethical Committee of Shanghai Tenth People's Hospital (approval number SHSY‐IEC‐15‐18), which was in accordance with the *Declaration of Helsinki*. Consent forms were also signed by patients. All experimental procedures involving animals were in accordance with the standard of *the Guide for the Care and Use of Laboratory animals* published by the National Institutes of Health.

### 2.2 Study objective

110 cases of HCC biopsy specimens were collected at the Shanghai Tenth People's Hospital and confirmed by pathological diagnosis. None of the patients received prior radiotherapy and chemotherapy. 46 cases were over 65 years old. There were 64 cases under 65 years old; 61 cases with tumour diameters <5 cm and 49 cases with tumour diameters over 5 cm. Of these, 33 paired of cases of HCC and adjacent tissues were randomly selected for further study.

### 2.3 Cell culture and transfection

Hep3B cell line (BNCC289780) was obtained from BN Bio (Beijing, China). MHCC97H cell line (YS‐ATCC087) was obtained from Shanghai YSRIBIO industrial co., LTD. (Shanghai, China). Human embryonic liver cells HHL5 were obtained from Wuhan Weikesaisi Technology Co., Ltd. (Wuhan, China). Human amniotic epithelial cells (HAECs) were obtained from Mingzhou Bio (Ningbo, China). Human umbilical vein endothelial cells (HUVECs) were obtained from Keygen BioTECH (Nanjing, China). Hep3B, MHCC97H and HHL5 were cultured in Dulbecco's Modified Eagle Medium (DMEM) (Gibco, Carlsbad, CA, USA) supplemented with 10% foetal bovine serum (FBS; Thermo Fisher Science, Inc, Waltham, MA, USA), 100 U/mL penicillin and 100 µg/mL streptomycin. HAECs and HUVECs were cultured in M‐199 medium supplemented with endothelial cell growth supplement: 100 U/mL penicillin and 100 µg/mL streptomycin. For the induction of angiogenesis, HUVECs or HAECs were stimulated by condition medium (50% supernatant from HepG3 + 50%M‐199 medium) after confluency reached 60%‐80% and normal HUVEC was as control. 3 × 10^5^ cells were plated in a 6‐well plate and transfected with indicated plasmid at 50% confluence by using Lipofectamine 2000 Transfection Reagent (11668‐019, Invitrogen, Carlsbad, California, USA) by following the instructions from the manufacturer. Opti‐MEM (51985042, Gibco, Gaitherburg, MD, USA) was mixed with 4μg indicated plasmids or Lipofectamin2000 for 5 minutes at room temperature and were then mixed in a two‐regent well for 20minutes before being added to a 6‐well plate. After transfection, cells were cultured at 37°C in a saturated humidity atmosphere containing 95% air and 5% CO_2_; the medium was replaced after 6 hours, and lysates were collected after 48 hours.

### 2.4 Immunohistochemistry (IHC)

Formalin‐fixed paraffin‐embedded 5‐μm tissue sections were incubated at 60°C for 30 minutes and then deparaffinized in xylenes, rehydrated through a graded series of alcohol concentrations for 5 minutes and rinsed by water for 2 minutes. After antigen retrieval by 1mM Tris‐EDTA (pH = 8.0) was performed, all sections were rinsed with phosphate buffer saline (PBS) for three times and left to block at room temperature in 3% H_2_O_2_‐methanol for 10 minutes, followed by the coating of antibodies at room temperature for overnight at 4℃. Afterwards, the sections were rinsed three times in 0.1% PBST, incubated with an Enhancer buffer (PV‐9000, Zsbio, Beijing, China) for 20 minutes and incubated with enzyme‐labelled anti‐mouse/rabbit secondary antibodies (PV‐9000, Zsbio) for 30 minutes. Subsequently, the proteins were subjected to diaminobenzidine (DAB) development and left to counterstain with haematoxylin for 1 minute. After the differentiation and addition of blue coloration, the sections were dehydrated by gradient concentration ethanol, transparentized and sealed by neutral resins. Immunohistochemistry images were obtained using an upright microscope (BX53, OLYMPUS, Japan) and analysed and scored by experienced pathologists. Primary antibodies used: KDM5A (1:400, Abcam, Cambridge, UK, ab217292) and CD31 (1:400, Abcam, ab9498).

### 2.5 Scratch assay

Each group of cells were seeded in a 6‐well plate as 2.5 × 10^4^/cm^2^. After cells were cultured for 24 hours, a scratch was made through the centre of each well using the 100 µL sterile pipette tip. The scratch was observed and imaged at the start and 24 hours following the scratch using a microscope. Image J software (1.48; National Institutes of Health) was used to measure the migrated distance. Cell migration capacity was evaluated by comparing scratch width. Rate of wound healing = (0‐width – 24‐width)/0‐width × 100%. Each group was set up with independent triplicates.

### 2.6 3‐(4,5‐dimethylthiazol‐2‐yl)‐5(3‐carboxymethonyphenol)‐2‐(4‐sulfophenyl)‐2H‐tetrazolium (MTS) assay

5 × 10^3^/wells MHCC97H and Hep3B cells were seeded in a 96‐well plate. Cells were transfected with indicated plasmids after being cultured for 24 hours. 20 μL MTS buffer (K300‐500, Amyjet Scientificlnc, China) was added to each well and incubated for 2‐4 hours after cells were transfected for 24 hours. The absorbance value was measured at 450 nm. Five duplicated wells were set for each group. Cell survival rates = (optical density (OD)^EXP^ − OD^Empty^/OD^Control^ − OD^Empty^)%.

The experiment was repeated 3 times.

### 2.7 Transwell assay

Cells were resuspended using the DMEM supplemented with 10 g/L bovine serum albumin (BSA) and adjusted to the final density of 1 × 10^4^ cells/mL. The Transwell chamber was put into a 24‐well plate, and the upper chamber surface of the bottom membrane of the Transwell chamber was coated with Matrigel (40111ES08, Yeasen BioTechnologies co., Ltd., Shanghai, China) at the ratio of 1:8 and air‐dried overnight at 4°C. After routine digestion, cells were resuspended in a culture medium, adjusted to a cell density of 1 × 10^5^ cells/mL, and 200 μL of the cell suspension was added to the top of the Transwell chamber covered with Matrigel (BD, San Jose, CA, USA). The lower chambers were supplemented with 600 μL medium containing 20% FBS. The Transwell chamber was collected following 24 hours of incubation at 37°C, where the Matrigel and cells were removed with a cotton swab, and subjected to fixation and then stained by crystal violet dissolved in methanol. Stained cells were counted in five random fields per well using an inverted microscope (XDS‐800D; Caikon, Shanghai, China). The invasive cells were counted. Triplicates were set in each group. This experiment was repeated for at least 3 times.

### 2.8 Matrigel^TM^ pseudo‐tube formation

Matrigel (Shanghai Shanran Biological Technology Co., Ltd., Shanghai, China) was placed in a refrigerator at 4℃ overnight to be melted into yellow gel‐like fluid. A total of 70 μL yellow gel‐like fluid was added to pre‐cooled 96‐well plate by pre‐cooled micropipette, followed by incubation at 37°C for solidification. After cells were transfected for 48 hours, they were starved for 1 hour and resuspended in DMEM to prepare for cell suspension. Cell suspension (1 × 10^5^ cells/mL) was seeded into pre‐coated 96‐well plate and added with medium of different groups. The cell plate was then incubated at 37°C for 18 hours. Each group was set up with independent triplicates. Three contiguous fields of pictures were taken by an upright microscope (×100) (Leica, Weztlar, Germany), and the number of intact capillary lumens surrounded by cells was analysed by Image J software. This experiment was repeated for at least 3 times.

### 2.9 Reverse transcription quantitative polymerase chain reaction (RT‐qPCR)

Total RNA content was extracted from tissue samples and cells using a Trizol kit (Invitrogen). RNA was then reversely transcribed into cDNA according to the instructions of a Reverse Transcription kit (K1622, Fermentas Inc, Ontario, USA). The stem‐loop method was used to reverse transcribe miR‐433 and its RT primer: 5'‐GTCGTATCCAGTGCAGGGTCCGAGGTATTCGCACTGGATACGACGCCACA–3'. With cDNA as a template, RT‐qPCR was reacted using TaqMan MicroRNA Assay and TaqMan® Universal PCR Master Mix. RT‐qPCR was subsequently performed according to the instructions of the TaqMan Gene Expression Assays protocol (Applied Biosystems, Foster City, CA, USA). U6 was used as the internal reference for miR‐433, and β‐Actin was used as the internal reference for mRNA. The 2^−ΔΔCt^ method was employed to calculate the ratio of the relative expression of a target gene in the experimental group to that of the control group with the following formula: ΔΔCt = ΔCt _experimental group_ − ΔCt _control group_ and ΔCt = Ct _target gene_ − Ct _internal reference_. Three independent experiments were conducted. Primers are listed in Table 1.

**TABLE 1:** Primer sequences used for RT‐qPCR

	Sequence
miR‐433	F 5‐ CGATCATGATGGGCTCCT ‐3
	R 5‐ GTGCAGGGTCCGAGGT ‐3
KDM5A	F 5‐AGCCGAGTTGGGAGGAGTT ‐3
	R 5‐ TGGACTCTTGGAGTGAAACGA‐3
FXYD3	F 5‐GGCCAGAAGTCCGGTCA‐3
	R 5‐ AACGGTCCTCCACCCAATTTC‐3
β‐Actin	F 5‐ TCACCCACACTGTGCCCATCT ‐3
	R 5‐CAGCGGAACCGCTCATTGCC ‐3
U6	F 5‐ CTCGCTTCGGCAGCACA ‐3
	R 5‐AACGCTTCACGAATTTGCGT ‐3

Note

microRNA‐433, miR‐433; KDM5A, Lysine demethylase 5A; FXYD Domain‐Containing Ion Transport Regulator 3, FXYD3.

#### 2.9.1 Western blot

Cells were lysed by radio immunoprecipitation assay (RIPA) lysis (R0010, Solarbio, Beijing, China), and the lysates were quantitated by BCA protein assay kit (GBCbio, Guangzhou, China). 40 µg protein sample was separated using sodium dodecyl sulphate polyacrylamide gel electrophoresis that had been electro‐transferred onto polyvinylidene fluoride (PVDF) membranes and probed with primary antibodies overnight at 4°C. Immunoblots were visualized with goat anti‐rabbit Immunoglobulin G (IgG; 1:2000, ab97051, Abcam) or goat anti‐mouse IgG (ab205719, 1:2000, Abcam) and enhanced chemiluminescence detection reagents and were captured under the Image Quant LAS 4000C (GE, USA General Electric Company, Schenectady, NY, USA) microscope. Primary antibodies used: KDM5A (Abcam, ab70892, 1:1000), FXYD3 (Abcam, ab205534, 1:1000), p‐p85 (Abcam, ab182651, 1:1000), p85 (CST, #4257 1:1000), p‐AKT (Abcam, ab81283, 1:1000), AKT (Santa Cruz, CA, USA, sc‐5298, 1:1000) and β‐Actin (Santa Cruz, sc‐8432, 1:1000).

#### 2.9.2 Dual luciferase assay

FXYD3 was predicted to be directly targeted by miR‐433 according to http://starbase.sysu.edu.cn/agoClipRNA.php?source. Dual luciferase assay was conducted to validate the binding affinity and the effect of miR‐433 overexpression on the FXYD3 3’UTR activity. Synthesized wild‐type (WT) or binding sites mutant (Mut) 3’UTR of FXYD3 fragments were introduced to the pMIR‐reporter vector (Beijing Huayueyang Biotechnology Co., Ltd., Beijing, China) by endonuclease sites: SpeI and Hind III. The complementary sequence mutation site of the seed sequence was designed on the FXYD3 WT 3'UTR, and the target fragment was inserted into the pMIR‐reporter reporter plasmid using T4 DNA ligase after restriction endonuclease digestion. Correctly sequenced WT‐ or Mut FXYD3 construct was cotransfected with miR‐433‐ or NC‐mimic in HEK‐293T cells (Beinuo Biological Technology Co., Ltd., Shanghai, China). Cell lysates were collected after cells were left to transfect for 48 hours. Luciferase activity was measured in Glomax20/20 luminometer (Promega) by Luciferase Detection Kit (K801, Biovision, Milpitas, CA, USA) by following the instructions provided by the manufacturer. Each group was set up with independent triplicates. This experiment was repeated for at least 3 times.

#### 2.9.3 Chromatin immunoprecipitation (ChIP)‐qPCR

ChIP assay was performed by using EZ‐Magna ChIP Kit (EMD Millipore, Billerica, MA, USA). Hep3B and MHCC97H cells were crosslinked by 4% formaldehyde and incubated with 0.125 M Glycin to crosslink DNA and protein. Next, cells were lysed and subjected to sonication to obtain 500 ~ 1000bp chromatin fragments. The lysates were incubated with primary antibodies Rabbit IgG (ab171870, Abcam; NC), anti‐KDM5A (ab70892, Abcam) and anti‐H3K4me3 (ab12209, Abcam) (IgG as negative control, H3K4me3 or KDM5A). The precipitated DNAs were analysed by RT‐qPCR. According to the predicted binding sites of KDM5A, the ChIP primers were designed as below: F: 5'‐GTTTCGCAGAGATAGCTTGGAG–3', R: 5'‐GGGGTGAAACTGAACAAGAACG‐3'.

#### 2.9.4 Enzyme‐linked immunoassay (ELISA)

ELISA was performed using human VEGF Quantikine PharmPak kit for VEGF‐A (R and D systems, USA), by following the manufacturer's instructions. 100 μL of CMs was added to 96‐well microwells and incubated for 2 hours at room temperature. After washing with PBS, 100 μL of biotin‐conjugate was added and washed. Next, 100 μL of streptavidin‐HRP was added to each well and kept for 1 hour at room temperature, and 100 μL later mixed and incubated for 30 minutes. The reaction was terminated by the addition of 100 μl of terminating solution. Colour density was read at 450 nm using a microwell reader (BioTek, Winooski, VT, USA).

#### 2.9.5 5‐Ethynyl‐2′‐Deoxyuridine (EdU) assay

A total of 5 × 10^3^ cells were seeded in a 96‐well plate. After 6 hours, 100 μL fresh medium containing 50 μmol\L Edu was added onto the plate and incubated at 37°C for 2 hours. After incubation, cells were fixed by 4% paraformaldehyde for 20 minutes, de‐crosslinked by 2 mg/mL glycin twice, incubated with PBST for 10 minutes and incubated with 100 µL Apollo staining solution for 30minutes. Afterwards, cells were treated with Hoechst33342 for 30 minutes in a dark room and washed by 0.5% Triton X. Lastly, three random views of the cells were observed under a fluorescence microscope. Cell number was counted by Image‐pro plus 6.0 software. Red fluorescence was represented Edu positive cells. Edu positive rates = (N_positive_/total cell number) %. This experiment was repeated for at least 3 times.

#### 2.9.6 Fluorescence in situ hybridization (FISH)

For FISH investigation, the frozen sections were incubated using the 100 nmol\L miR‐433 FISH probe in 2 × SSC and 20% formamide at 37°C for 1 hour. After sections were washed for three times by DEPC‐PBS, these sections were stained with 0.5 μg/mL DAPI and then washed three times for CLSM imaging (Nikon, C2, Tokyo, Japan).

#### 2.9.7 Xenograft tumour mouse model

Four‐week‐old female BALB/c nude mice (n = 20, weighing 18‐25 g) were obtained from SLAC Laboratory Animal Co., Ltd. (Hunan, China). Mice were given a standard diet and raised in a specific pathogen‐free animal facility. MHCC97H cells stably transfected with siRNA (si)‐negative control (NC) (NC for si‐KDM5A), si‐KDM5A, si‐NC (NC for si‐KDM5A) or si‐KDM5A) were subcutaneously injected into the dorsal flanks of mice. Bidimensional tumour measurements (the product of the longest diameter and its longest perpendicular diameter for each tumour) were recorded every 2 days after infection during the span of 2 weeks. Mice were killed after 30 days, and the tumours were excised for further experimental procedures. Tumour volume (mm^3^) was measured with calipers and calculated as (B^2^ × A)/2, where B is width and A is length.

#### 2.9.8 Statistical analysis

Data were presented as the mean ± standard deviation from at least three independent experiments performed in triplicates. Data between HCC tissues and adjacent tissues were analysed using a paired *t* test. Statistical comparisons were performed using an unpaired *t* test when only two groups were compared, whereas Tukey's test‐corrected one‐way analysis of variance (ANOVA) was used when comparisons of more than two groups were made. Variables were analysed at different time points using Bonferroni‐corrected repeated measures of ANOVA. The correlation of measurements was yielded with Pearson's correlation analysis. All statistical analyses were completed with SPSS 21.0 software (IBM, Armonk, NY, USA), with two‐tailed *P* < .05 as a level of statistical significance.

## 3 RESULTS

### 3.1 KDM5A was highly expressed in HCC and is correlated with a poor prognosis in patients with HCC

**FIGURE 1:**
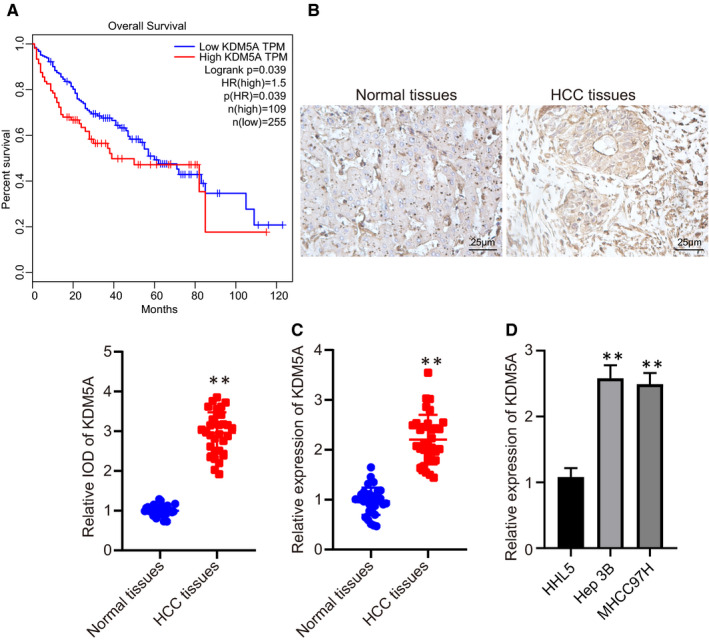
KDM5A was significantly up‐regulated in HCC tissues and was negatively correlated with overall survival rates. A, the correlation between KDM5A expression and overall survival rates analysed by GEPIA. B, KDM5A expression levels in HCC tissues determined by IHC, ×400. C, KDM5A expression in HCC tissues and normal tissues determined by RT‐qPCR, N = 110. D, KDM5A expression in Hep3B, MHCC97H and HHL5 determined by RT‐qPCR, N = 3. *P < .05; **P < .01, compared to that of normal tissues. Data were shown as the mean ± standard deviation. Statistical comparisons were performed by Tukey's test‐corrected one‐way ANOVA when more than two groups were compared. The experiment was repeated 3 times

A previous study has revealed an up‐regulated expression of KDM5A in HCC.^20^ With the attempt to further validate its expression in HCC, we performed prognosis analysis using GEPIA website, revealing that KDM5A was negatively correlated with the overall survival rate of patients with HCC (Figure 1A). Therefore, KDM5A is correlated to a poor prognosis of patients with HCC. Furthermore, the expression of KDM5A was observed to be significantly increased in HCC tissues compared to that of benign tissues by analysing biopsy specimens through IHC and RT‐qPCR (Figure 1B,C) (*P* < .05). KDM5A expression level was much higher in HCC cell lines compared to that of normal liver cell lines (Figure 1D) (*P* < .05). KDM5A was highly expressed in both HCC tissues and cell lines and is negatively correlated with HCC patient overall survival rates.

### 3.2 KDM5A silencing suppresses HUVEC angiogenesis, HCC proliferation, migration and invasion capabilities

**FIGURE 2:**
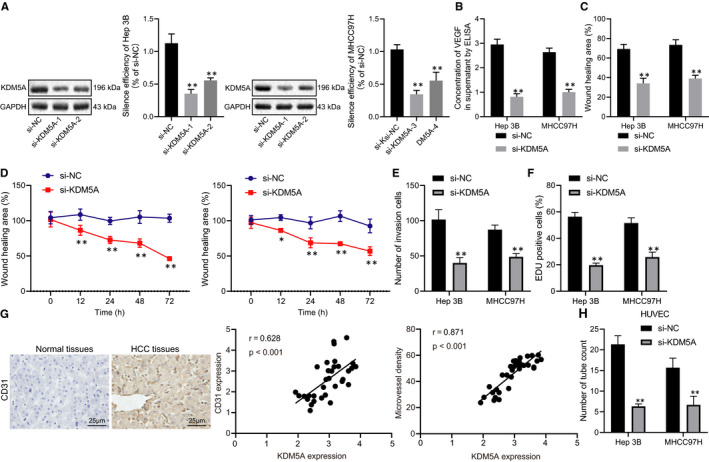
Depletion of KDM5A suppressed the proliferative, migrative, invasive and angiogenic capacities of HCC cells. A, silencing efficiency of independent KDM5A siRNAs in Hep3B and MHCC97H cells determined with RT‐qPCR. B, VEGF expression in the supernatant of Hep3B and MHCC97H cells measured by ELISA. C, effect of KDM5A silencing on the migrative capacities of Hep3B and MHCC97H cells determined by scratch assay. D, effect of KDM5A silencing on the cell viability of Hep3B and MHCC97H cells determined by MTS. E, effect of KDM5A silencing on the invasion capacities of Hep3B and MHCC97H cells determined by transwell assay. F, effect of KDM5A silencing on proliferation determined by EDU assay. G, CD31 expression levels in HCC biopsy specimens determined by IHC. H, the effect of media from KDM5A silenced Hep3B and MHCC97H cells on the angiogenesis of HUVECs determined by pseudo‐tube formation assay. *P < .05; **P < .01, compared to si‐NC. Data were shown as the mean ± standard deviation. Statistical comparisons were performed by Tukey's test‐corrected one‐way ANOVA when more than two groups were compared. The experiment was repeated 3 times

To investigate the role of KDM5A in HCC angiogenesis, we silenced KDM5A in Hep3B and MHCC97H found si‐KDM5A‐1 achieving the best knock down efficiency in Hep3B cells, while si‐KDM5A‐3 was suitable for MHCC97H cells (Figure 2A). We determined the expression of VGEF by ELISA and found that the silencing of KDM5A had significantly down‐regulated the expression of VEGF in Hep3B and MHCC97H cells (Figure 2B) (*P* < .05). In addition, wound healing assay revealed that inhibiting KDM5A had significantly suppressed Hep3B and MHCC97H migration capabilities (Figure 2C,) (*P* < .05). MTS assay indicated that KDM5A silencing inhibited the viability of these two HCC cell lines (Figure 2D) (*P* < .05). Meanwhile, transwell assay demonstrated that the depletion of KDM5A led to the down‐regulation of invasion capacity of Hep3B and MHCC97H cells (Figure 2E) (*P* < .05). Inhibited proliferative capabilities were found in Hep3B and MHCC97H cells after inhibiting KDM5A (Figure 2F) (*P* < .05). CD31 has been reported to play a vital role in angiogenesis (*P* < .05). We detected CD31 expression levels in hepatoma biopsy specimens and found CD31 was highly expressed in HCC tissues. Pearson's correlation analysis revealed that the level of CD31 expression was positively correlated with KDM5A (Figure 2G). Angiogenesis assay suggested that the angiogenesis capacity of HUVECs was significantly suppressed when cultured in condition media from KDM5A‐silenced Hep3B and MHCC97H cells (Figure 2H) (*P* < .05). Taken together, our data revealed that silencing KDM5A could inhibit the progression of HCC by suppressing angiogenesis.

### 3.3 KDM5A suppresses miR‐433 expression

**FIGURE 3:**
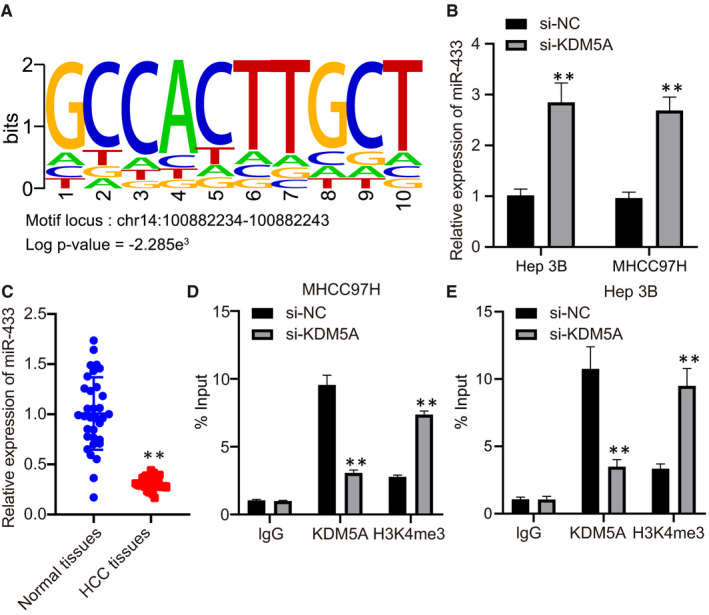
KDM5A suppressed miR‐433 transcription. A, binding site of KDM5A predicted by ChIPBase. B, miR‐433 expression levels after depleting KDM5A determined by RT‐qPCR. C, miR‐433 expression in HCC tissues and normal tissues determined by RT‐qPCR. D, the enrichments of KDM5A and H3K4me2 on miR‐433 promoter region in Hep3B cells determined by ChIP assay. E, the enrichment of KDM5A and H3K4me2 on miR‐433 promoter region in MHCC97H cells determined by ChIP assay. *P < .05; **P < .01, compared to si‐NC or adjacent tissues. Data were shown as the mean ± standard deviation. Statistical comparisons were performed by Tukey's test‐corrected one‐way ANOVA when more than two groups were compared. The experiment was repeated 3 times

Previous reports have demonstrated KDM5A suppressing downstream genes expression by binding to the promoter of these genes and demethylating H3K4me3.^30^ According to the ChIPBase database, KDM5A could bind to the promotor region of miR‐433 and its binding region was in chr14: 100 882 337‐100 882 570, of which its binding site was analysed, e indicating that KDM5A might transcriptionally regulate miR‐433 (Figure 3A). RT‐qPCR results revealed that miR‐433 level was significantly up‐regulated after inhibiting KDM5A (Figure 3B). To further confirm our hypothesis, we analysed the expression of miR‐433 in HCC tissues and found that the expression of miR‐433 was down‐regulated in HCC tissues compared with that of adjacent tissues (Figure 3C) (*P* <.05). Moreover, ChIP assay revealed KDM5A could directly bind to the promotor region of miR‐433 and H3K4me3 enrichment in the promotor region of miR‐433 was dramatically increased after KDM5A silencing, which indicated KDM5A had suppressed the expression of miR‐433 by demethylating H3K4me3 through binding to its promoter (Figure 3D,E). Hence, KDM5A suppressed miR‐433 transcription by demethylating H3K4me3 in HCC cells.

### 3.4 KDM5A promotes the proliferative, migrative, invasive and HUVEC angiogenic properties of HCC cell angiogenesis by suppressing miR‐433

**FIGURE 4:**
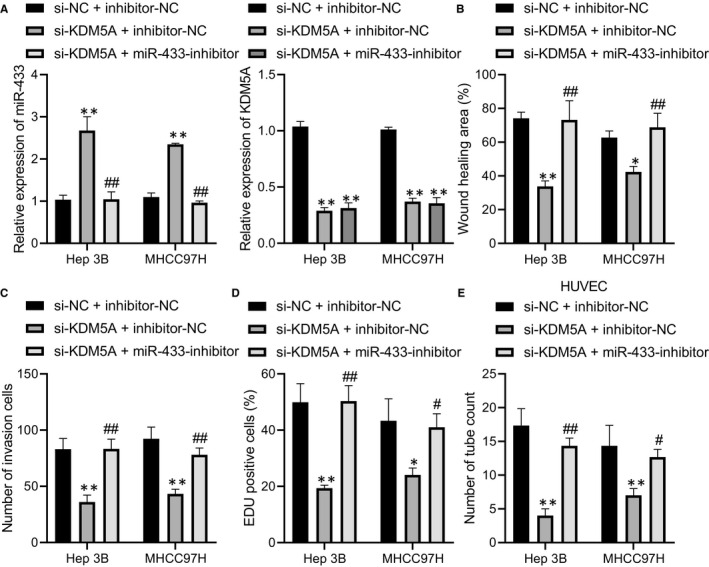
Depletion of KDM5A up‐regulated miR‐433 to suppress HCC angiogenesis and progression. A, the expression levels of miR‐433 and KDM5A after restoration of miR‐433 in KDM5A‐silenced Hep3B and MHCC97H cells determined by RT‐qPCR. B, the effect of miR‐433 restoration on the migrative capacity of KDM5A silenced Hep3B and MHCC97H cells determined by scratch assay. C, the effect of miR‐433 restoration on invasive capacity of KDM5A silenced Hep3B and MHCC97H cells determined by transwell assay. D, the effect of miR‐433 restoration on proliferative capacity of KDM5A silenced Hep3B and MHCC97H cells determined by EDU assay. E, the effect of miR‐433 restoration on angiogenesis of KDM5A silenced Hep3B and MHCC97H cells determined by pseudo‐tube formation assay. *P < .05; **P < .01, compared to si‐NC+ inhibitor‐NC. #*P* < .05; ##*P* < .01, compared to si‐KDM5A+ inhibitor‐NC. Data were shown as the mean ± standard deviation. Statistical comparisons were performed by Tukey's test‐corrected one‐way ANOVA when more than two groups were compared. The experiment was repeated 3 times

To investigate the functions of KDM5A‐miR‐433 axis in the progression of HCC, we silenced KDM5A and miR‐433 in Hep3B and BEL‐7404 and found that miR‐433 was significantly up‐regulated after KDM5A was silenced and its expression was dramatically reduced after the transfection of the miR‐433 inhibitor (Figure 4A) (*P* < .05). Scratch test and Transwell assay revealed that the restoration of miR‐433 rescued the reduced migrative and invasive capacities of Hep3B and BEL‐7404 caused by KDM5A depletion (Figure 4B,C) (*P* < .05). Similarly, miR‐433 silencing alleviated the proliferative capacities after depletion of KDM5A (Figure 4D). The reduced angiogenic capacity induced by depleting KDM5A was also restored by miR‐433 depletion (Figure 4E). Thus, KDM5A promoted the proliferative, migrative, invasive and angiogenic properties of HCC cells by down‐regulating miR‐433.

### 3.5 miR‐433 negatively targets FXYD3

**FIGURE 5:**
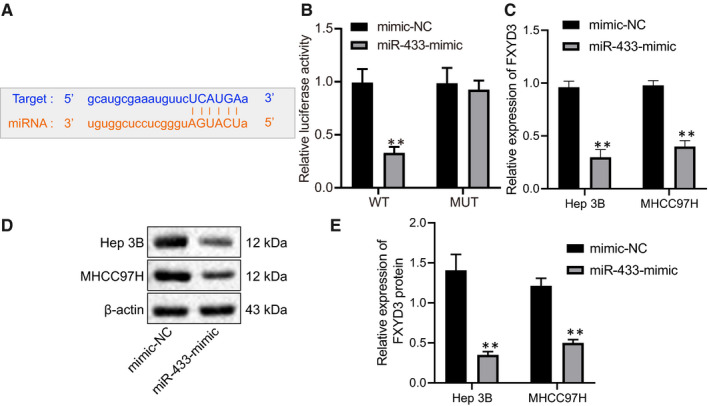
miR‐433 directly targeted FXYD3. A, binding sites of miR‐433 on FXYD3 predicted by TargetScan. B, target relationship validated by dual luciferase assay. C, the expression of FXYD3 after overexpressing miR‐433 determined by RT‐qPCR. D, FXYD3 expression after overexpressing miR‐433 determined by Western blot. E, quantification of FXYD3 protein levels normalized to β‐actin. *P < .05; **P < .01, compared to mimic‐NC. Data were shown as the mean ± standard deviation. Statistical comparisons were performed by Tukey's test‐corrected one‐way ANOVA when more than two groups were compared. The experiment was repeated 3 times

A previous report demonstrated FXYD3 was highly expressed in HCC tissues and is negatively correlated with overall survival rates.^12^ On the contrary, miR‐433 had been reported to suppress the progression of HCC.^12^ Therefore, we assumed miR‐433 might regulate the progression of HCC by targeting FXYD3. We found miR‐433 might target FXYD3 and the potential binding sites on FXYD3 were identified by Starbase (Figure 5A). Based on this, we performed dual luciferase assay and found that overexpressing miR‐433 had significantly repressed the luciferase activity of FXYD3‐3’UTR‐WT, but not its mutant form which indicated miR‐433 directly binding onto and suppressing FXYD3 expression (Figure 5B) (*P* < .05). RT‐qPCR and Western blot demonstrated that the overexpression of miR‐433 had significantly down‐regulated FXYD3, both at mRNA and protein levels in MHCC97H and Hep3B cells (Figure 5C‐E) (*P* < .05). Therefore, miR‐433, a downstream target of KDM5A, directly targeted and suppressed the expression of FXYD3.

### 3.6 Inhibition of KDM5A inhibits FXYD3‐PI3K‐AKT axis to repress the proliferative, migrative, invasive and angiogenic properties of HCC

**FIGURE 6:**
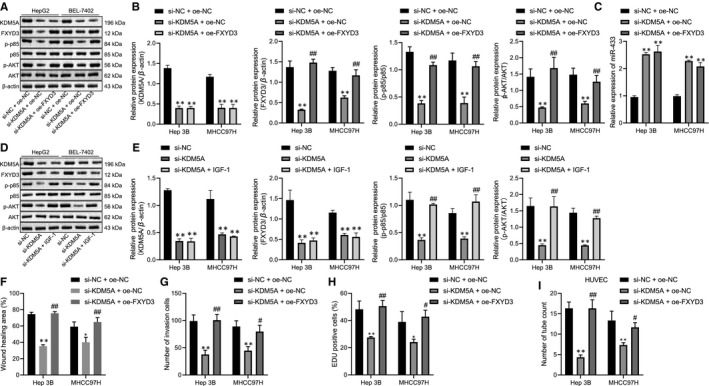
Depletion of KDM5A repressed FXYD3‐PI3K‐AKT axis to suppress the proliferative, migrative, invasive and angiogenic capacities of HCC cells by up‐regulating miR‐433. A, KDM5A, FXYD3, p‐p85, p85, p‐AKT and AKT detected by Western blot after restoration FXYD3 in KDM5A silenced Hep3B and MHCC97H cells. B, quantification of (A) related protein levels. C, after restoration FXYD3 in KDM5A silenced Hep3B and MHCC97H cells, miR‐433 expression levels were determined by RT‐qPCR. D, after KDM5A depleted cells treated with IGF‐1, KDM5A, FXYD3, p‐p85, p85, p‐AKT and AKT detected by Western blot. E, quantification of (D) related protein levels. F, the effect of FXYD3 restoration on migrative capacity of KDM5A silenced Hep3B and MHCC97H cells determined by scratch assay. G, the effect of FXYD3 restoration on invasive capacity of KDM5A silenced Hep3B and eMHCC97H cells determined by transwell assay. H, the effect of FXYD3 restoration on proliferative capacity of KDM5A silenced Hep3B and MHCC97H cells determined by EDU assay. I, the effect of FXYD3 restoration on angiogenesis of KDM5A silenced Hep3B and MHCC97H cells determined by pseudo‐tube formation assay. *P < .05; **P < .01, compared to si‐NC + oe‐NC. #*P* < .05; ##*P* <.01, compared to si‐KDM5A + oe‐NC. Data were shown as the mean ± standard deviation. Statistical comparisons were performed by Tukey's test‐corrected one‐way ANOVA when more than two groups were compared. The experiment was repeated 3 times

FXYD3 had been reported to activate PI3K‐AKT signalling and aberrant activated PI3K‐AKT signalling could promote HCC angiogenesis.^12^ To further confirm whether KDM5A regulated FXYD3‐PI3K‐AKT signalling by miR‐433, we restored the expression of FXYD3 after silencing KDM5A in Hep3B and MHCC97H. We found out that FXYD3 was significantly down‐regulated; p‐p85 and p‐AKT protein expression levels returned to basal levels after restoring FXYD3 expression (Figure 6A,B) (*P* < .05). Furthermore, restoration of FXYD3 did not affect up‐regulation of miR‐433 caused by silencing KDM5A (Figure 6C) (*P* > .05). We also treated KDM5A‐silenced cells with IGF‐1 (Akt agonist) and found that IGF‐1 treatment reactivated PI3K‐AKT signalling without affecting the expressions of KDM5A and FXYD3 (Figure 6D,E) (*P* < .05).

Subsequently, we performed scratch assay and found that overexpressing FXYD3 rescued the reduced migrative, invasive and proliferative capacities of HCC cells caused by KDM5A silencing (Figure 6F‐H) (*P* < .05). The angiogenic capacity of HUVECs, cultured in condition media from KDM5A silenced cells, was significantly reduced, while the restoration of FXYD3 after KDM5A silencing reversed this phenotype (Figure 6L‐M) (*P* < .05). Taken together, inhibition of KDM5A repressed FXYD3‐PI3K‐AKT signalling to suppress HCC progression by up‐regulating miR‐433.

### 3.7 Silencing of KDM5A down‐regulates FXYD3‐PI3K‐AKT axis to inhibit HCC tumorigenesis *via* miR‐433 up‐regulation in vivo

**FIGURE 7:**
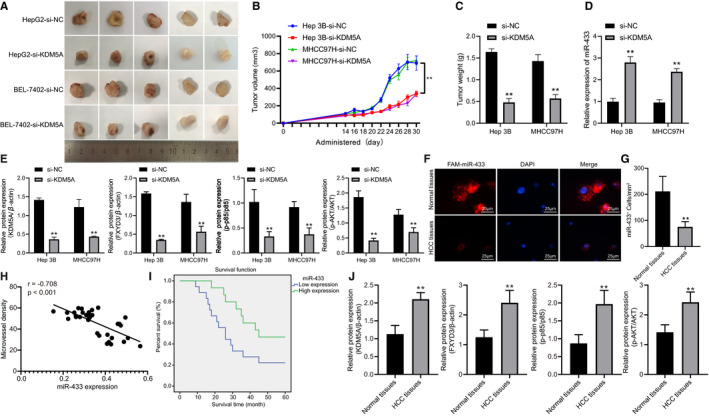
KDM5A regulates miR‐433‐FXYD3‐PI3K‐AKT axis to promote HCC tumorigenesis. A, Representative images of xenograft tumours after subcutaneous injection for 30 days. B, volume of xenograft tumours after subcutaneous injection at different time points. C, weights of xenograft tumours after subcutaneous injection for 30 days. D, expression of miR‐433 in mice xenografted with tumours determined by RT‐qPCR. E, protein levels of KDM5A, FXYD3, p‐p85/p85 and p‐AKT/AKT in xenograft tumours detected by Western blot, N = 5. F, expression of miR‐433 in biopsy specimens determined by FISH. G, quantification of FISH, N = 33. H, Pearson's correlation analysis of correlation between miR‐433 and angiogenesis (microvessel density) in clinical samples. I, Kaplan‐Meier survival analysis of correlation between miR‐433 and overall survival rates, N = 33. J, protein levels of KDM5A, FXYD3, p‐p85, p85, p‐AKT and AKT in clinical samples detected by Western blot, N = 33. *P < .05; **P < .01, compared to Normal tissues. Data were shown as the mean ± standard deviation. Statistical comparisons were performed by Tukey's test‐corrected one‐way ANOVA when more than two groups were compared. The experiment was repeated 3 times

To investigate the effect of regulating miR‐433‐FXYD3‐PI3K‐AKT signalling by KDM5A on HCC tumorigenesis, Hep3B and MHCC97H cells were subjected to subcutaneous injection in nude mice after being transfected with KDM5A shRNA. Based on this, we found that silencing KDM5A had significantly suppressed HCC tumorigenesis, tumour volume and tumour weight (Figure 7A‐C) (*P* < .05). We detected the presence of miR‐433, FXYD3, p‐AKT and p‐p85 expressions in tumour tissues, which demonstrated that KDM5A silencing led to significant increase in miR‐433 and reduction in expression levels of FXYD3, p‐AKT and p‐p85 in tumour tissues (Figure 7D,E) (*P* < .05). miR‐433 expression in HCC tissues was validated by FISH. Results revealed that miR‐433 was significantly down‐regulated in clinical HCC tissues (Figure 7F,G). Meanwhile, we also found that miR‐433 was negatively correlated with angiogenesis (Figure 7H). Moreover, the overall survival rates of patients with HCC were positively correlated with the expression of miR‐433 (Figure 7I) (*P* < .05). On the other hand, the protein levels of KDM5A, FXYD3, p‐AKT and p‐p85 were much higher in clinical HCC tissues compared to that of normal tissues (Figure 7J). In conclusion, KDM5A promoted HCC tumorigenesis in vivo by regulating miR‐433‐FXYD3‐PI3K‐AKT signalling.

## 4 DISCUSSION

Angiogenesis describes the formation of new blood vessels from pre‐existing ones, which is a complicated physiological process forms the basis for solid tumour growth.^31^, ^32^ VEGF has been regarded as the major pro‐angiogenic regulator and a drug target for anti‐angiogenic therapy.^33^ More importantly, a previous report mentioned that HCC belongs to one of the highly vascularized solid tumours and is accompanied with hypervascularity and vascular abnormalities.^4^ The efficiency of anti‐angiogenesis therapy on the treatment of HCC is very limited.^7^ Increasing evidence demonstrates that miRNAs are critical for the regulation of angiogenesis.^34^ Accumulated evidence revealed that miR‐433 acts as a tumour suppressor miRNA.^11^ Consistent to the previous studies, our findings revealed that miR‐433 was dramatically down‐regulated in hepatocellular tissues and in vitro cell lines. Furthermore, overexpressing miR‐433 significantly suppressed VEGF expression and angiogenesis in vitro, as well as HCC tumour formation in vivo.

Numerous studies revealed that epigenetic restructuring is tightly intertwined with cancer initiation and development,^35^ as the alternation of the epigenetic modifications in tumour cells might lead to resistance to typical therapeutic strategies.^36^ As to the role of epigenetic factors in favouring angiogenesis, a recent report demonstrated EZH1, a methyltransferase, to promote angiogenesis by catalysing H3K27me3.^37^ In‐depth investigation of the molecular mechanism of down‐regulating miR‐433 in HCC, we noticed that KDM5A, a demethylase for H3K4me2/3, is predicted to a directly bind to the miR‐433 promoter region. In the present study, KDM5A regulated the expression of miR‐433 by removing H3K4me3 on its promoter region. However, the role of KDM5A in HCC angiogenesis remains largely unknown. We analysed HCC biopsy specimens and found KDM5A expression was significantly increased in HCC tissues, but was negatively correlated with miR‐433, the results of which were consistent with a previous study.^20^ The expression of KDM5A is negatively correlated with the overall survival rates of patients with HCC. In vitro assays demonstrated that silencing KDM5A up‐regulates miR‐433 to reduce migrative and invasive capacities, growth and suppress angiogenesis. Moreover, the up‐regulation of miR‐433 is correlated with the proliferative, migrative, invasive and HUVEC angiogenic capacities in HCC cells. miR‐433 was found to be down‐regulated, which indicates a poor outcome and limited overall survival rates.^12^ Interestingly, overexpressing miR‐433 inhibited focus formation in vivo and inhibits the oncogenic phenotype development of HCC.^12^

In the subsequent study, we found that miR‐433 negatively targeted FXYD3. FXYD3, majorly found in the breast, colon, stomach and prostate, is able to interact with Na, K‐ATPase b subunit and regulates the transport properties of Na, K‐ATPase.^38^ FXYD3 has been reported to be highly expressed in several types of cancers, including breast cancer, and is related to the survival rate and metastasis.^24^ FXYD3 has been reported to be a clinical diagnosis marker of HCC. Based on this, restoration of FXYD3 expression rescues the inhibited oncogenic phenotype of HCC caused by KDM5A silencing. The up‐regulated expression level of FXYD3 in HCC is associated with HCC clinicopathological characteristics, serving as a potential prognostic marker for HCC.^39^ Furthermore, our data revealed that FXYD3 promotes the progression of HCC by activating PI3K‐AKT signalling. FXYD3 has been reported to be highly expressed in diverse cancer tissues and its aberrant up‐regulation contributed to the progression of breast cancer by activating PI3K‐AKT signalling.^23^, ^24^ Aberrantly activated PI3K‐AKT is frequently expressed in various cancers,^40^ in which the continuous activation of PI3K‐AKT contributes to the progression of cancer, e indicating a bad clinical outcome.^26^ PI3K‐AKT re‐activation promotes Her2 target therapy in breast cancer.^41^ Combination treatment with AKT inhibitor overcomes Lapatinib treatment resistance.^42^ PIK3CA mutation or amplification of HER2 or other RTK receptors are the leading causes of aberrantly activated PI3K‐AKT.^27^, ^28^ Besides, abnormal up‐regulation of PI3K‐AKT upstream signalling also contributes to the aberrant activation of PI3K‐AKT axis.^23^

## 5 CONCLUSIONS

**FIGURE 8:**
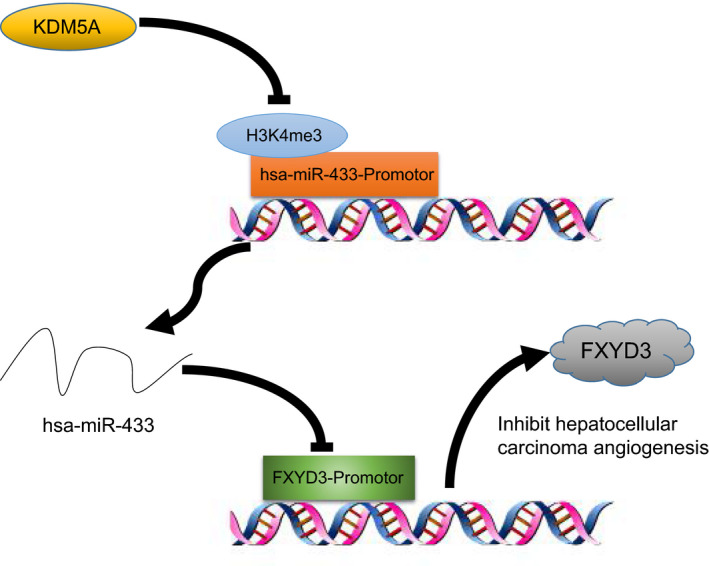
A diagram illustrating the molecular pathway of KDM5A in HCC. Notes: KDM5A suppresses miR‐433 expression at a transcriptional level to promote the FXYD3‐PI3K/AKT axis, which further accelerates the angiogenesis of HCC

Our data suggested that KDM5A directly binds to the promotor region of miR‐433 and suppresses its expression by removing H3K4me3. The down‐regulation of miR‐433 up‐regulates FXYD3 levels in HCC tissues, which leads to the aberrant activation of PI3K‐AKT signalling, contributing to the angiogenic process of HCC (Figure 8). Importantly, selective inhibitor of KDM5A has been developed. Based on our studies, we hypothesized that KDM5A inhibitor treatment may be beneficial to traditional anti‐angiogenic therapy. Therefore, future experiments on this speculation are warranted to increase the efficiency of anti‐angiogenic therapy in HCC.

## CONFLICT OF INTEREST

The authors declare that they have no conflict of interest.

## AUTHOR CONTRIBUTIONS

YSM, TMW, YSL, HD, MMF and FY: Conceptualization. YSM, HMW, YS and LPG: Data Curation. HMW, YS, LL and LLT: Formal analysis. YSL, MMF and JBL: Investigation. HD, MMF and JBL: Methodology. LPG and DF: Project administration. LL and DF: Resources. TMW, BQ and FY: Software. QFZ and CCL: Supervision. BQ and PYW: Validation. GRW and ZJW: Visualization. TMW, YSL, HD, YSM and LLT: Writing‐original draft. BQ, PYW, GRW, ZJW, QFZ, CCL and DF: Writing‐review and editing.

## Data Availability

The data sets generated and/or analysed during the current study are available from the corresponding author on reasonable request.
